# Design of EM-artifact-free earphone based on the photoacoustic effect

**DOI:** 10.1016/j.pacs.2020.100214

**Published:** 2020-12-09

**Authors:** Bengi Derya Musdal, Mustafa Kurt

**Affiliations:** a Department of Physics, Çanakkale Onsekiz Mart University, 17100 Çanakkale, Turkey; b Department of Electronic Engineering, Çanakkale Onsekiz Mart University, 17100 Çanakkale, Turkey

**Keywords:** Photoacoustic effect, Molecular infrared modes, Audible sound, Photonics

## Abstract

Electromagnetic interactions between conventional earphones and the electroencephalography (EEG) electrodes used for analyzing brain waves give rise to efficiency problems in neurophysiological studies of auditory perception. Currently used speakers and headphones are electromagnetic devices based on strong magnets. In spite of intensive use of such systems, there has been no effective way to eliminate the electromagnetic artifacts produced by such audio transmitting devices to date. The ability for transferring audible sounds without the use of electromagnetic devices that can affect the EEG signal would open up many innovative possibilities in Audio Technologies. Audible sound transfer over long distances is possible by the photoacoustic effect. In such studies, the modulated optical signal can be converted into an audible signal arising from the absorption of the light energy of relevant molecules. In this study, we propose an earphone based on the photoacoustic effect, and calculated the dB SPL (Sound Pressure Level) values for a spherical cell filled with olive pomace. By the use of the method of Diebold and Westervelt, we theoretically calculated the sound pressure levels for our cell and determined a 60 dB SPL at a sound frequency of 1000 Hz for our preliminary earphone design.

## 1 Introduction

Almost all of the commercially available audio transducers, such as earphones or headphones, are based on the working principle of moving coils to produce magnetic fields up to 4.5 Tm (*Bl* product) [1]. In auditory-neuro perceptual experiments, electroencephalography (EEG) is used for measuring the electro-activity of the human brain. One can thus see what happens in the brain when people (for example musicians or sound engineers) hear and perceive different musical sounds. When earphones and EEG are used simultaneously, earphones cause electromagnetic (EM) artifacts on the EEG electrodes corresponding the EEG signals, and such artifacts are often misinterpreted by the neurophysiologist. The main working principle of EEG is to sense EM waves produced by currents between neurons. EM waves emerging from neurons are very weak, so that they can be missing in the EM noise coming from external devices (e.g. from earphones or cables) [[2], [3], [4]]. Some types of earphones (e.g. piezoelectric earphones, electrostatic earphones, electromagnetic shelled earphones or normal earphones used with acoustic tubes) interact less with EEG electrodes, but can be very expensive or not efficient enough for EEG applications [3,4]. One example of such inefficient earphones in EEG applications are normal earphones used in conjunction with acoustic tubes. In this case there is a time delay between the sound signal and the brain response because of the path difference in the tube [3]. This time delay often leads to a misinterpretation of the EEG signal.

In contrast to the availability of EM interaction-free earphones with high fidelity, no headphones based on the photoacoustic (PA) working principle have been reported. EM-artifact free earphones can be constructed from the principles of the PA effect and “PA communications” [5]. The motivation of this work is to develop an audio system that does not interfere with EEG electrodes, and to develop earphones adaptable to EEG and at the same time avoid interaction with its electrodes. Such a development should be applicable to other platforms such as Functional Magnetic Resonance Imaging (fMRI). In this manuscript we present theoretical calculations and a preliminary conceptual design of a PA earphone in this context.

## 2 Theory

The generation of acoustic waves by modulated optical radiation is usually referred to as PA or optoacoustics (OA) [6]. Alexander Graham Bell’s Photophone was the starting point of PA and photothermal (PT) phenomena [7]. Bell transmitted sound waves by collimating sunlight, but his achievement was too limited owing to insufficient technical apparatus at that time. After the invention of the photophone, most scientists focused on the creation of new methods and approaches concerning the PA effect for audible sound and on other applications [[5], [6], [7], [8]]. Sullenberger et al. improved a new technique in 2019 for transferring a sound wave by use of laser light [5]. When the emission wavelength of the laser matches the absorption line of an appropriate molecule, the molecular bonds vibrate in synchrony with the modulation frequency of the laser beam. If this modulation is in the audible sound frequency range, re-production of audible sound based on the PA effect is possible [[5], [6], [7], [8]]. Sullenberger et al. has calculated the theoretical values of the sound pressure for the case of H_2_O molecules by use of Tam's results [5,6]. By this means they experimentally demonstrated that their new approach is applicable to the transmission of a sound wave with a laser beam [5].

PT phenomena are generally based on PA generation, but some other phenomena may also be used, such as the absorption of molecular vibrational energy in the IR region of the electromagnetic spectrum. The energy of the laser beam in this case is absorbed by molecular bonds in an intrinsic medium. If the length of molecular bonds produces a match to the laser wavelength (or wavenumber), the molecules vibrate in resonance. This vibration causes an expansion in volume of the molecules. Under isobaric conditions, enhanced pressures can occur at the surface of a spherical region occupied by the molecules. If the relaxation time of these molecules is lower than the duty cycle of the optical signal, a sound wave can be produced at a desired frequency in the audible region of the sound spectrum. The sound pressure level (SPL) in dB is dependent on the molecular species, the geometric shape of the exposed medium, and on the intensity and wavelength of the laser beam [5]. Audible sound level ranges are between 0 and 120 dB SPL. In order to produce these values, the pressure changes on the surface of the sphere must reach 20 Pa. In psychoacoustics, hearing is not flat in terms of frequency response, but depends on the loudness, which is a psychological term describing the magnitude of an auditory sensation. Equal loudness curves define the inherent necessity of equal hearing, known as Fletcher-Munson curves. The reference level of Phon in this curve is taken as 0 Phon for 0 dB SPL at a 1000 Hz sound frequency [9]. The dB SPL and corresponding pressure values at any frequency also can be obtained from the Fletcher-Munson curves. The calculated dB SPL and pressure levels vs. the pure tones of equal hearing between 0–100 Phon values for different sound frequencies are shown in Table 1 [9].

**Table 1:** Loudness levels (Phon) vs. dB SPL values corresponding Pressure (Pa) values [9].

**Loudness** **Level** **(Phon)**	**Frequency (Hz) :**	**100**	**500**	**1000**	**5000**	**10000**
**0**	dB SPL	38	6	**0**	−5	8
	Pressure (mPa)	1.59	0.04	****0.02****	0.01	0.05
**20**	dB SPL	52	25	**20**	18	30
	Pressure (mPa)	7.96	0.36	****0.2****	0.16	0.63
**40**	dB SPL	62	42	**40**	40	52
	Pressure (mPa)	25.18	2.52	****2****	2	7.96
**60**	dB SPL	72	61	**60**	60	73
	Pressure (mPa)	79.62	22.44	****20****	20	89.34
**80**	dB SPL	83	80	**80**	78	91
	Pressure (mPa)	282.51	200	****200****	158.87	709.63
**100**	dB SPL	100	101	**100**	95	107
	Pressure (mPa)	2000	2244.04	****2000****	1124.68	4477.44

Westervelt et al. calculated the expansion of an absorbing medium in the shape of a droplet exposed to a modulated laser beam [10,11]. When the radius of the laser beam is smaller than the droplet radius, the thermal conduction can be neglected, and the wave equation for the pressure can be defined as

(1) $${□}^{2}p=\nabla^{2}p-\frac{1}{c_{0}^{2}}\frac{\partial^{2}p}{\partial t^{2}}=-\frac{\text{β}}{C_{p}}\frac{\partial H}{\partial t},$$

where $c_{0}$ is the velocity of the sound for the medium, β is the logarithmic coefficient of thermal expansion, *C_p_* is the specific heat per unit mass, and *H* is the absorbed energy per unit volume and time [10,11]. The modulated laser beam loss energy per unit distance and time according to

(2) $$H=\text{α}I_{0}e^{-\text{α}z-i\text{ω}t},$$

where *I*_0_ is the laser intensity, α is the optical absorption coefficient of the molecule, and ω is the modulation frequency (ω=2πf) [10]. By inserting Eq. (2) into Eq. (1), Westervelt et al. found that the pressure value for a sphere is given by

(3) $$p=-\frac{i\text{α}\omega \beta I_{0}}{4\pi C_{P}}e^{-i\text{ω}t}\int e^{-\alpha z'}\frac{e^{ik\left|\vec{r}-\vec{r'}\right|}}{\left|\vec{r}-\vec{r'}\right|}d^{3}\vec{r'}.$$

where $\vec{r}$ is the vector position of the observation point and $\vec{r'}$ is the vector from a point on the sphere to the observation point [10]. Assuming that r>>ω/c₀α², the pressure integral becomes [10]

(4) $$p=-\frac{i\text{α}\omega \beta S_{0}I_{0}}{4\pi rC_{P}}e^{ikr-i\text{ω}t}\int_{0}^{\infty }e^{-\alpha z'-ikz'\cos\theta }dz'.$$

Westervelt et al. stated that the attenuation coefficient affects the exposed medium in two ways: 1) the effect of the laser beam expansion is smaller than that of energy absorption in the case of a high attenuation coefficient and 2) the attenuation length is greatly increased in the case of a small attenuation coefficient [10]. In the first case, the thermal expansion of the molecule is higher and therefore to fill the sphere we chose a molecule having a high attenuation coefficient at the laser wavelength. Diebold and Westervelt described the pressure of a spherical droplet exposed to the PA effect for different boundary conditions [11]. They derived a pressure expression for the case of a smaller beam radius than the sphere radius by use of results from their previous study. If the density and sound velocity of the outer medium is equal to the inner medium, and assuming that the medium is optically thin, the pressure becomes

(5) $$P\left(q\right)=-\frac{i\alpha \beta I_{0}c_{0}a}{C_{p}\hat{r}}\left(\frac{\sin q-q\cos q}{q^{2}}\right)e^{-iq\left(\hat{\tau }-1\right)},$$

where a is the radius of the sphere, $\hat{r}=r/a$ is the dimensionless distance from the center of the sphere, $q=\omega a/c_{0}$ is the dimensionless frequency (or wave vector) and $\hat{\tau }-1=(c_{0}/a)(t-r/c_{0})$ is the dimensionless retarded time with respect to propagation from the center of the sphere [11]. For the condition of q≪1 and r≈a, we expand the sine and cosine functions up to the second degree by use of a Taylor Series, and Eq. (5) can be rewritten as:

(6) $$P=-i\frac{2\pi }{3C_{P}}\alpha \beta I_{0}a^{2}f$$

which is the sound pressure where f is the frequency of the sound.

## 3 Method

**Fig. 1:**
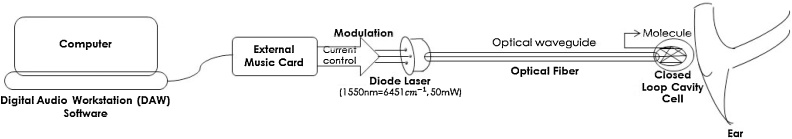
Preliminary conceptual photoacoustic based earphone design.

In this study, we present a preliminary conceptual design of a PA earphone and calculate the pressure arising from the PA effect for a spherical cell filled with olive pomace. Our conceptual design for the PA earphone consists of a sound source, a laser that can be modulated, an optical fiber and a spherical absorption cell (Fig. 1). Digitized audible sound is generated by Digital Audio Workstation (DAW) software. Sound samples with pure tones at specific frequencies, pink noise including a full spectral audible range and various musical sounds are prepared in order to obtain a balanced and coherent signal with the Fletcher-Munson curves by the use of ProTools Free, DAW software. In the conceptual design configuration of this earphone based on the PA effect, digital audio samples coming from DAWs are transmitted to a diode laser input by an external sound card. Electronic output of the external sound card is used as a bias for the diode laser (1550 nm =6451 cm^−1^, 50 mW). The amplitude of the laser beam is modulated in accordance with the bias voltage and therefore the duty cycle of the laser modulation must be taken into consideration in the pressure calculations. Atalar studied the PA effect for a liquid enclosed in a cylindrical cell and expressed the PA signal is driven in cw and pulse modulation schemes analytically and demonstrated his result experimentally [12]. He predicted that the conversion loss of the transducer is 10 dB in the case of high frequency continuous wave modulation (1 MHz) [12]. The duty cycle of the signal in our design is shorter than Atalar’s and therefore we expect a low loss for the PA signal in the cell, and less interference between the main signal and the reflected signal from the wall of the sphere. Thus, our conceptual earphone design is expected to possess high fidelity. The volume of the sound can be adjusted with the laser power. The modulated laser beam is transmitted with an optical fiber cable to the absorption cell placed in a closed loop enclosure for safety. The spherical absorption cell contains the molecule, which has the same absorption lines as the laser wavelength in order to maximize the sound pressure level. The molecular structure of the medium in the cell is crucial for our design. As mentioned before, the peak absorption wavelength of the molecule should be overlapped to the laser wavelength, and the molecule should have a high expansion coefficient and a small heat capacity. The IR absorption bands of some molecular bounds are reported in the literature [[13], [14], [15]]. Molecules having RNH2 (amide group) or NH (first overtone) are the most appropriate for our conceptual earphone design [13]. In particular, studies in the literature show that olive pomace and an articular cartilage with polystyrene has a high absorbance at 1550 nm wavelength [14,15]. The beginning of the acoustic meatus for humans has generally a 10−13 mm radius [3]. Making use of this information, we calculated the pressure for a 10 mm diameter sphere filled with olive pomace by use of Eq. (6). Olive pomace is a byproduct of olive oil production, and contains both olive kernel and olive oil. The inherent structure of raw olive pomace is a mixture of solid and liquid [15,16]. A weak PA signal is produced when such a cell is filled with a light-absorbing liquid [17]. On the contrary, it can be expected that a strong PA signal can occur in the case of a cell which is filled with a light-absorbing soft solid material. Some thermophysical properties of the olive pomace are quite close to olive oil [18,19]. Taking the main thermophysical properties of olive pomace to be equal to that of olive oil, we obtain the attenuation coefficient, the expansion coefficient and the heat capacity as α (1550 nm)≈1, β = 0.00070 K^−1^ and *C*_p_ = 1970 Jkg^−1^ K^−1^, respectively [15,[18], [19], [20], [21]]. We determined the theoretical pressure from Eq. (6) for a sphere filled with olive pomace to be 8.3 mPa at 1000 Hz and 165.98 mPa at 20000 Hz. These pressure levels correspond to almost 50 dB SPL and 60 dB SPL, respectively, which correlate with the Fletcher Munson curves. If the laser power is increased it should be possible to reach 80 dB SPL.

## 4 Conclusions

In conclusion, we have developed an EM artifact-free PA earphone design, in which a modulated laser beam is guided through to a sphere filled with olive pomace by means of an optical fiber. The optical fiber takes the place of an electrical cable. In addition, we do not make use of coils or magnets, unlike conventional earphones. Our EM artifact-free conceptual earphone design has the potential to be used for EEG applications. Theoretically, 80−100 dB SPL values can be obtained when suitable components are incorporated in our proposed design. Our new type of conceptual earphone design can not only be used in neuro-perceptual studies with EEG in or other schemes in a controlled manner, but also can be used for listening to music with high fidelity. Such high fidelity will be tested in the experimental phase of future studies.

## Author statement

Bengi Derya Muşdal’s current research interests include photoacoustics, audio perceptual studies, EEG-sound modules with no interfere electromagnetically, photoacoustic-based speakers and earphones.

Mustafa Kurt’s current research interests are in lasers, photonics, liquid crystals and acoustic frequency converting by piezoelectric sensors.

## Declaration of Competing Interest

The authors declare that there are no conflicts of interest.
